# Mobile Apps for Common Noncommunicable Disease Management: Systematic Search in App Stores and Evaluation Using the Mobile App Rating Scale

**DOI:** 10.2196/49055

**Published:** 2024-03-12

**Authors:** Khang Jin Cheah, Zahara Abdul Manaf, Arimi Fitri Mat Ludin, Nurul Huda Razalli, Norﬁlza Mohd Mokhtar, Sawal Hamid Md Ali

**Affiliations:** 1 Center for Healthy Ageing and Wellness, Faculty of Health Sciences, Universiti Kebangsaan Malaysia, Kuala Lumpur, Malaysia; 2 Faculty of Medicine, Universiti Kebangsaan Malaysia, Kuala Lumpur, Malaysia; 3 Electronic and Systems Engineering, Faculty of Engineering and Built Environment, Universiti Kebangsaan Malaysia, Selangor, Malaysia

**Keywords:** mHealth apps, mobile health, health apps, chronic diseases, self-management, app quality, apps, app, application, applications, quality, MARS, Mobile App Rating Scale, mHealth, chronic, review methods, review methodology, review of apps

## Abstract

**Background:**

The success of mobile apps in improving the lifestyle of patients with noncommunicable diseases through self-management interventions is contingent upon the emerging growth in this field. While users of mobile health (mHealth) apps continue to grow in number, little is known about the quality of available apps that provide self-management for common noncommunicable diseases such as diabetes, hypertension, and obesity.

**Objective:**

We aimed to investigate the availability, characteristics, and quality of mHealth apps for common noncommunicable disease health management that included dietary aspects (based on the developer’s description), as well as their features for promoting health outcomes and self-monitoring.

**Methods:**

A systematic search of English-language apps on the Google Play Store (Google LLC) and Apple App Store (Apple Inc) was conducted between August 7, 2022, and September 13, 2022. The search terms used included *weight management*, *obesity*, *diabetes*, *hypertension*, *cardiovascular diseases*, *stroke*, and *diet*. The selected mHealth apps’ titles and content were screened based on the description that was provided. Apps that were not designed with self-management features were excluded. We analyzed the mHealth apps by category and whether they involved health care professionals, were based on scientific testing, and had self-monitoring features. A validated and multidimensional tool, the Mobile App Rating Scale (MARS), was used to evaluate each mHealth app’s quality based on a 5-point Likert scale from 1 (inadequate) to 5 (excellent).

**Results:**

Overall, 42 apps were identified. Diabetes-specific mHealth apps accounted for 7% (n=3) of the market, hypertension apps for 12% (n=5), and general noncommunicable disease management apps for 21% (n=9). About 38% (n=16) of the apps were for managing chronic diseases, while 74% (n=31) were for weight management. Self-management features such as weight tracking, BMI calculators, diet tracking, and fluid intake tracking were seen in 86% (n=36) of the apps. Most mHealth apps (n=37, 88%) did not indicate whether there was involvement of health professionals in app development. Additionally, none of the apps reported scientific evidence demonstrating their efficacy in managing health. The overall mean MARS score was 3.2 of 5, with a range of 2.0 to 4.1. Functionality was the best-rated category (mean score 3.9, SD 0.5), followed by aesthetics (mean score 3.2, SD 0.9), information (mean score 3.1, SD 0.7), and engagement (mean score 2.9, SD 0.6).

**Conclusions:**

The quality of mHealth apps for managing chronic diseases was heterogeneous, with roughly half of them falling short of acceptable standards for both quality and content. The majority of apps contained scant information about scientific evidence and the developer’s history. To increase user confidence and accomplish desired health outcomes, mHealth apps should be optimized with the help of health care professionals. Future studies on mHealth content analysis should focus on other diseases as well.

## Introduction

Globally, noncommunicable diseases (NCDs) account for 74% of all deaths yearly (41 million people), which highlights the global health burden [1]. In Malaysia, the latest National Health and Morbidity Survey reported that two-thirds of the Malaysian population have at least 1 of 3 common NCDs, namely diabetes, hypertension, or hypercholesterolemia, and about half of the population (50.1%) are overweight or obese [2]. There are barriers to care access, delivery, and self-management for the management of NCDs, such as being unable to visit a clinic in a timely manner and long consultation waiting times [3], prompting policy makers to improve the health care system. The introduction of mobile health (mHealth) technology presents an opportunity for patient self-monitoring, helping health care providers personalize the management of patients [4] and increase cost-effectiveness throughout health management [5].

According to a definition by the World Health Organization, mHealth is the use of mobile and wireless devices such as mobile phones, tablets, and personal digital assistants to support health care management [6]. In the rapidly growing mHealth app market, the presence of these apps could facilitate the health care management system. A recent review of the literature revealed that researchers have recognized that mHealth could be an effective tool in chronic disease management [4] and improve patients’ self-management behavior [7]. A growing body of research demonstrates the health benefits of mHealth interventions for patients with NCDs in terms of enhancing patient self-monitoring and health outcomes in NCDs such as type 2 diabetes [8], obesity [9], and cardiovascular diseases [10].

Self-management is crucial in the daily management of chronic diseases to improve quality of life and reduce management costs [11]. However, poor self-management among patients with chronic disease has been observed [12]. Technologies such as mHealth have the potential capacity to empower patients requiring support in their self-management efforts. A review by Cruz-Ramos et al [10] demonstrated that many mHealth apps for cardiovascular diseases support self-management features such as medical advice, reminders, and self-monitoring notifications [10]. Moreover, research has also found that self-monitoring of weight and dietary intake is associated with positive outcomes for weight loss [13]. As the key to person-centered care, mHealth apps enhance self-management for chronic conditions by providing personalized goal setting, active reminders, social interaction, and support [14]. Hence, mHealth apps could help build decision-support systems that bridge the gap between self-management and conventional health care management.

As of 2022, there were nearly 2.67 million mobile apps available on the Google Play Store. Of these, more than 130, 000 apps were health care or health and fitness apps [15]. The number of mHealth apps available on the Google Play Store and Apple App Store continues to grow [16-18]. Globally, it was estimated that 6.6 billion individuals own a smartphone, and the number is expected to grow to 7.7 billion by the year 2027 [19], which allows mHealth technology to be more accessible to individuals. However, caution must be taken regarding this growth, as the evidence related to its efficacy and benefits for chronic health disease management is not well identified.

Previous research on the perception and usability of mHealth apps for NCD management has demonstrated a growing interest in user-centric health-tracking mobile apps among the population with chronic illness [20,21]. An empirical study conducted to predict patients’ intentions to continue using mHealth services as part of self-managing their chronic conditions revealed that the participants had high intentions to continue the use of mHealth services [22]. The use of mHealth apps is highly encouraged, as it has been linked to higher rates of health-promoting behavior among people with chronic medical conditions [23]. Self-management using mHealth apps could be a part of health management, as people living with NCDs have the autonomy to take responsibility for their health. Relevant content analysis studies have been carried out in different geographical areas [24-26], but limited studies have been conducted among Southeast Asian countries. As such, there is an emerging need to bridge this research gap by initiating more content analysis studies in Malaysia to contribute to more holistic development of mHealth apps.

This paper provides a review of the current landscape of mHealth apps, with an emphasis on common chronic disease management. Understanding trends in mHealth apps and their relevant features will benefit users in developing informed decisions, as well as help health care providers improve the quality of mHealth. To make better-informed decisions, the reliability of currently existing mHealth apps should be explored. This study aims to describe mHealth apps available in conventional app stores for common NCDs, determine their health categories, explore the features they focus on, identify neglected areas, and evaluate their quality using the Mobile App Rating Scale (MARS) assessment tool.

## Methods

This review involved a systematic search of apps available in mobile phone app stores. The protocol adhered to the 5-step framework outlined by Arksey and O’Malley [27], which includes (1) identifying the research question; (2) identifying relevant apps; (3) selecting apps; (4) charting the data; and (5) collating, summarizing, and reporting the results.

### Identifying the Research Question

This review aimed to answer the following questions: “What are the available mHealth apps in the Malaysian market for common NCDs?” “What are the app features available in the mHealth apps for NCDs in Malaysia?” and “What is the quality of the mHealth apps for NCDs in Malaysia?”

### Identifying Relevant Apps

The search was conducted from July 7, 2022, to August 14, 2022, on the Apple App Store (Apple Inc) and Google Play Store (Google LLC) using the search terms *weight loss*, *obesity*, *diabetes*, *hypertension and cardiovascular diseases*, *stroke*, *weight management,* and *diet*. The search terms were identified using appropriate Medical Subject Headings terms as well as the free text of keywords. The selected NCDs were chosen because they are among the most prevalent chronic diseases in Malaysia and around the world.

### Selecting Apps

To be eligible for inclusion in this review, the title and content of the identified mHealth apps were screened and filtered based on the descriptions provided by the app developer. An app met the inclusion criteria if it (1) was developed in English; (2) had self-monitoring feature(s); and (3) was developed for chronic disease management, namely obesity, type 2 diabetes, hypertension, and cardiovascular disease. An app was excluded based on the following criteria: it was a heart rate tracker, exercise tracker, or game; or it was a medicine-delivery, appointment-based, recipe-sharing, or research study app.

### Charting the Data

The data were extracted based on feature categories, which included (1) type of health management, (2) number of downloads, (3) country of the developers, and (4) app features. A data extraction table with the mHealth apps’ basic information, such as country of origin, name of app developer, and number of downloads, is shown in Table 1. The details of the mHealth apps’ features and characteristics are outlined in Multimedia Appendix 1.

**Table 1.** Overview of selected mHealth apps.

Name of app	Number of downloads	Availability of in-app purchase	Cost of in-app purchase (US $)	Country of app developer	App developer	Operating system
MyFitness Pal: Calorie Counter	100,000,000	Yes	0.76-0.70	US	MyFitnessPal, Inc	Android
Health & fitness tracker with calorie counter	5,000,000	Yes	0.65-2.90	India	DROID INFINITY	Android
Lifesum: Healthy eating & diet	10,000,000	Yes	3.95-50.30	Sweden	Lifesum	Android
Withings Health Mate	1,000,000	No	0.00	France	Withings	Android
Fitbit	50,000,000	Yes	7.20-290.70	US	Fitbit LLC	Android
HealthifyMe	10,000,000	Yes	7.65-72.10	Singapore	HealthifyMe	Android
Noom: Weight loss	10,000,000	Yes	0.76-142.10	US	Noom Inc	Android
Personal Health Monitor	100,000	No	0.00	Ukraine	Extrawest	Android
Life Extend: Healthy Habits	100,000	Yes	3.10-203.30	US	LifeOmic	Android
Qardio Heart Health	100,000	No	0.00	Canada	Qardio Inc	Android
FitTrack MyHealth: Track Scale	100,000	Yes	9.61-92.90	US	Fittrack	Android
Calorie counter by lose it!	10,000,000	Yes	4.25-161.75	US	FitNow Inc	Android
One Drop: Better Health Today	1,000,000	Yes	20.10-20.35	US	One Drop	Android
Calorie Counter - MyNetDiary	1,000,000	Yes	3.75-60.10	US	MyNetDiary.com	Android
Healthi: Personal Weight Loss	500,000	Yes	1.30-52.45	US	Sunshine Health Studios	Android
Health Diet Foods Fitness Help	500,000	Yes	2.20-21.90	India	RecoveryBull.com	Android
Unimeal: Healthy Diet&Workouts	100,000	Yes	5.50-95.10	Cyprus	Uniwell	Android
Health Click Away	10,000	Yes	3.10-21.85	US	HealthClickAway	Android
Possible-Nutrition Weight Loss	1,000,000	Yes	0.00	India	Truweight Wellness	Android
Health Club-Home workouts& Fitness-calorie tracker	50,000	No	0.00	—^a^	Health Club Group	Android
Smart Diet Planner weight loss	100,000	Yes	9.2-28.40	India	Appneurons Technologies Private Limited	Android
Heart Care Health & Diet Tips	10,000	Yes	2.2-21.90	India	RecoveryBull.com	Android
Calorie Counter + (Nutracheck)	1,000,000	Yes	1.85-36.10	England	Nutracheck	Android
Health Mate - Calorie Counter & Weight Loss App	500,000	Yes	0.90-5.69	India	PIXEL BYTES	Android
Doctor2u- One Stop Healthcare	500,000	Yes	2.20	Malaysia	BP Healthcare Group	Android
BookDoc- Go Active Get reward	500,000	Yes	1.75-17.50	Malaysia	BookDoc	Android
Health Pal - Fitness, Weight loss coach, Pedometer	1,000,000	Yes	1.65	India	Digit Grove	Android
Creda- manage chronic condition	100,000	Yes	1.75-18.40	US	KnowYourMeds Inc	Android
Mhealth	—	No	0.00	—	mutifun LLC	iOS
my Mhealth	—	No	0.00	England	my mhealth	iOS
Zero: Fasting & Health Tracker	—	Yes	10.10-69.95	US	Zero Longevity Science Inc	iOS
Lose Weight at Home in 30 Days	—	Yes	5.25-54.65	Hong Kong	ABISHKKING LIMITED	iOS
BodyFast Intermittent Fasting	—	Yes	3.95-61.2	Germany	BodyFast GmbH	iOS
BetterMe: Health Coaching	—	Yes	4.60-36.70	Cyprus	BetterMe Limited	iOS
Weight Loss Running by Slimkit	—	Yes	8.75-38.25	UK	MONTIBUS LTD	iOS
My Diet Coach - Weight Loss	—	Yes	2.20-8.75	US	Easy Tiger Apps LLC	iOS
Fitness Coach & Diet: FitCoach	—	Yes	9.20-57.95	Cyprus	A.L. AMAZING APPS LIMITED	iOS
Argus: Calorie Counter & Step	—	Yes	9.20-27.35	US	Azumio Inc	iOS
Speedoc - Care Comes to You	—	No	0.00	Singapore	Speedoc	iOS
Glucose Buddy Diabetes Tracker	—	Yes	3.70-54.65	US	Azumio Inc	iOS
DOC2US - Trusted Online Doctor	—	No	0.00	Malaysia	Doc2Us	iOS
Foodvisor - Nutrition & Diet	—	Yes	17.9-80.90	France	Foodvisor	iOS

a Not available (information was not found in app stores).

### Collating, Summarizing, and Reporting the Results

After obtaining the screening results, we performed a descriptive analysis, comparison, and functionality assessment based on the information provided by the app developers. In addition, we analyzed the mHealth apps’ category, as well as whether health care experts were involved, whether they were based on scientific testing, and whether they had self-monitoring, based on the description provided by the app providers. Additional information, such as star ratings and the presence of a privacy policy, was also tabulated. A star rating offers a quick overview of an app’s overall user satisfaction, making it a valuable component of quality assessment. The presence of a privacy policy reveals how an app manages user data, ensuring the protection of users’ private health information, which is crucial for informed decision-making.

### Quality Assessment of mHealth Apps

The quality of the mHealth apps was assessed using the validated MARS evaluation tool, which has demonstrated excellent internal consistency (α=.90) [28]. The MARS has 4 sections for objective evaluation: engagement (eg, the level of entertainment provided, interactivity, and appropriateness of app content), functionality (eg, app performance, ease of use, and navigation), aesthetics (eg, layout, graphics quality, and overall visual appeal), and information (eg, accuracy of the app description, source of information, and quality of information). The subjective quality evaluation section of the MARS subscale has 4 items. However, we excluded this section in this study as the aim was to assess the apps’ quality objectively. Each item was evaluated using a Likert scale with a score range from 1 (inadequate) to 5 (excellent). The overall quality score was calculated based on the mean scores for each of the 4 sections. A mean score of 3 was considered the minimum acceptable score, whereas a score greater than 4 of 5 was preferable. Before the evaluation, 2 authors independently used each of the apps and conducted the quality assessment in agreement with each other; disagreements were resolved through discussion with a third author.

## Results

### Search Results

**Figure 1.**
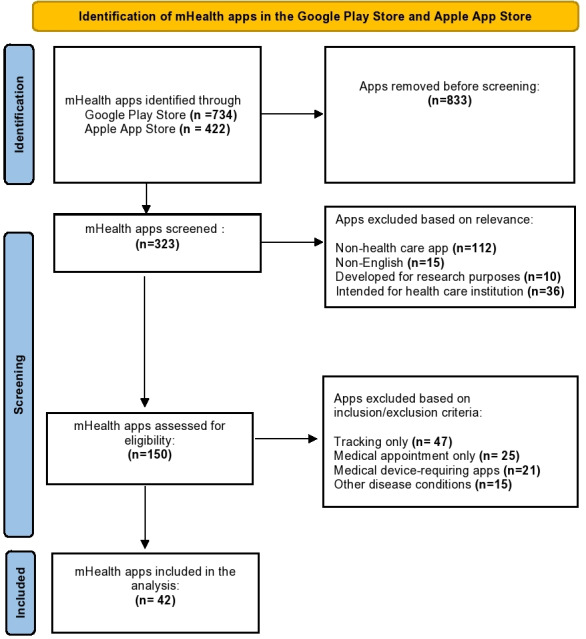
PRISMA (Preferred Reporting Items for Systematic Reviews and Meta-Analyses) flow diagram for the systematic search and selection of mobile health (mHealth) apps.

Our search found a total of 1156 apps through keyword retrieval from the Apple App Store and Google Play Store (Figure 1). After removing duplicates, 323 apps were screened; 150 apps met the inclusion criteria as apps focusing on selected chronic disease management (iOS: n=103; Android: n=47) and were included for eligibility assessment. Among them, 42 were included in this study for analysis.

### Sample Distribution and App Characteristics

A total of 42 apps (n=28 Android and n=14 iOS apps) were included in this review. Among these, 31 (74%) were for weight management while 11 (21%) were dedicated to chronic disease health management. The chronic diseases commonly targeted by the apps included cardiovascular disease (n=1, 2%), type 2 diabetes (n=3, 7%) and hypertension (n=5, 12%). However, there were 9 apps (21%) that did not specify which chronic diseases they targeted. Most of the apps received recent updates, in the year 2022. The general characteristics of the mHealth apps are described in Table 2. The download count is exclusive to Android apps, as this information was not obtainable for iOS apps.

**Table 2.** Sample distribution of chronic disease management apps (n=42).

Category		Apps, n (%)
Weight management		31 (74)
Type 2 diabetes mellitus		3 (7)
Hypertension		5 (12)
Cardiovascular disease		1 (2)
General NCDs^a^		9 (21)
**Year updated**		
	2020	1 (2)
	2021	5 (12)
	2022	36 (86)
**Downloads (Android only), n**		
	10,000-99,999	3 (7)
	100,000-999,999	12 (29)
	≥1,000,000	13 (31)
**Cost to download**		
	Free	42 (100)

a NCD: noncommunicable disease (this included hypertension, type 2 diabetes mellitus, and cardiovascular diseases).

As shown in Table 1, about 81% (n=34) of the apps provided in-app purchases for live-chat subscriptions, ad removal, and premium feature subscriptions ranging from weekly to yearly, among others. The price for each purchase was between US $0.66 to US $203.26. All the apps included in this review can be freely downloaded by users. Most of the apps (n=15, 36%) were developed in the United States, followed by India (n=7, 17%), Malaysia (n=3, 7%) and Singapore (n=2, 5%).

### Star Ratings and Privacy Policies of the Included mHealth Apps

Two apps on the Google Play Store and 6 apps on the Apple App Store received no reviews. The number of users (more than 1 million) who provided ratings for Android apps was significantly greater than the number for iOS apps. Most apps received a user rating of more than 4 stars of a total of 5. In this study, all the apps had a privacy policy. Table 3 shows the star ratings and privacy policies of the included apps.

**Table 3.** Star ratings and privacy policies of the included mobile health apps.

	Android^a^	iOS^b^
Star rating (1-5 stars), mean (SD)	4.3 (0.3)	4.6 (0.3)
Privacy policy, n (%)	28 (100)	14 (100)

a Apps without ratings: n=2.

b Apps without ratings: n=6.

### App Feature Assessment

The most common feature that was available on the mHealth apps was self-monitoring (n=35, 83%), which allows users to track their body weight, food intake, fluid intake, and other health indicators. Approximately 16 of 42 apps provided online consultation or personalized feedback via the app as well as goal-setting features (n=35, 83%). Meanwhile, there were no evidence-based apps that used scientific testing or multidisciplinary team involvement in app development, based on the descriptions provided by the developers. About 62% of apps (n=26) introduced at least 1 health care professional (eg, health coach, nutritionist, or dietitian) in the health management of the app. The overview of the functionality characteristics of the apps is presented in Table 4.

**Table 4.** Overview of functionality assessment of selected mobile health apps (n=42).

Components		Apps, n (%)	
		Yes	No
Multidisciplinary team involvement in app development		0 (0)	42 (100)
Health care professional involvement in app health management		26 (62)	16 (38)
**Self-monitoring features**			
	Overall	35 (83)	7 (17)
	Weight tracker (eg, BMI)	35 (83)	7 (17)
	Diet tracker	29 (69)	13 (31)
	Water intake tracker	16 (38)	26 (62)
	Step count tracker	10 (24)	32 (76)
	Exercise tracker	13 (31)	29 (69)
	Personalised feedback (eg, chat with doctor, nutritionist, health coach)	16 (38)	26 (62)
**Goal setting**			
Overall	35 (83)	7 (17)
Weight	35 (83)	7 (17)
Nutrient intake	16 (38)	26 (62)
Steps activity	9 (21)	33 (79)
	Exercise	5 (12)	37 (88)
**Medical condition monitoring**			
Overall	13 (31)	29 (69)
Blood pressure	9 (21)	33 (79)
Glucose	8 (19)	34 (81)
	Heart rate	7 (17)	35 (83)
Social support		15 (36)	27 (64)
Evidence-based testing		0 (0)	42 (100)

### App Quality Assessment

The average MARS score among the 42 apps was 3.2 of 5, with a range between 2.0 and 4.1. Of the 4 MARS domains, functionality scored the highest (3.9/5), followed by aesthetics (3.2/5), information (3.1/5), and engagement (2.9/5). There was a large gap in the scores of each subdomain, with the engagement score ranging from 1.6 to 4.0, functionality score ranging from 3.0 to 5.0, aesthetics score ranging from 1.0 to 5.0, and information score ranging from 1.7 to 5.0. The MARS functionality score had the smallest range, and the information score had the largest range. Table 5 shows the MARS subdomain ratings and the total mean score.

**Table 5.** App quality rating scores using the Mobile App Rating Scale (n=42).

Objective quality rating	Mean score (SD)	Minimum to maximum score
Engagement	2.9 (0.6)	1.6-4.0
Functionality	3.9 (0.5)	3.0-5.0
Aesthetics	3.2 (0.9)	1.0-5.0
Information	3.1 (0.7)	1.7-5.0
Total quality rating	3.2 (0.5)	2.0-4.1

## Discussion

### Principal Findings

Most NCD management apps in Malaysia lack scientific evidence of efficacy, do not involve multidisciplinary teams, and require significant feature improvements. This study provides a snapshot of current mHealth apps for common NCDs in 2022 that were primarily commercially based. It revealed that the mHealth apps varied in terms of features, functionality, and disease management. The most common category that the mHealth apps currently focus on is self-management of weight, followed by hypertension and type 2 diabetes. Some apps need to be purchased to access extended features, health management information, and medical advice.

Self-monitoring or self-management are crucial components of long-term chronic disease management. Self-management of chronic diseases has been reported to be related to positive health outcomes among patients [29]. Most of the mHealth apps (n=35, 83%) from this study incorporated self-monitoring features that allow the user to monitor their weight, blood pressure, blood glucose level, dietary intake, and fluid intake. The most common self-monitoring features found in the apps were weight trackers, food intake trackers, and step trackers. As technology has advanced, self-management through mHealth apps has been the subject of investigation. Systematic reviews in Korea [14] and the United States [12] examined their impact, shedding light on their potential role in achieving desirable health outcomes through self-management. This suggests that self-monitoring features in mHealth apps may be instrumental in achieving desirable health outcomes.

Some of the apps (n=15, 36%) incorporated social support features to allow users to engage with other users as well as health professionals. These social features could be key for users to continue engaging with the mHealth apps to improve their health. Social support has been shown to improve patients’ health and well-being, and this also applies to online social support networks [30]. Only 38% (n=16) of the apps offered communication with health care professionals via instant messaging or robotic automated message chat functions. These features are beneficial in the self-management of NCDs, as they allow the user to communicate with a health care professional and receive immediate feedback. There is evidence that 2-way communication between patients and health care professionals can improve health outcomes [31]; therefore, app developers should prioritize the inclusion of communication features and health care professionals in app management.

This study reveals a lack of evidence supporting the use of mHealth apps, with none of the apps reporting scientific evidence to indicate the effectiveness of their health management. Based on the star ratings, we cannot deny that mHealth apps could potentially help users improve their health outcomes. Given the fact that most apps on the Apple App Store (iOS) and Google Play Store (Android) did not provide evidence-based testing to prove their effectiveness [32], the app developers were able to make false or misleading claims about their apps. Moreover, health care providers are less likely to feel skeptical of the role of mHealth apps in health care management if the app is supported by research as clinical evidence [33].

The multidisciplinary team approach is a treatment domain that optimizes the health of patients with chronic diseases [34]. Promoting a multidisciplinary team approach is crucial for coordinating the health care system and aiding patients in self-management [35]. However, our research revealed that the majority of mHealth apps did not incorporate a multidisciplinary team to support health management. In this study, only 16% (n=7) of the apps indicated the participation of medical doctors, specialists, nurses, or dietitians. Our findings emphasize the need for multidisciplinary team involvement in health management by using reliable and high-quality mHealth apps. mHealth apps have the potential to function as proactive disease self-management tools [36], meeting diverse needs through collaborative efforts with multidisciplinary teams within the realm of mHealth technology.

The apps in the app stores generally had high star ratings, ranging from 4.3 to 4.6 of 5 stars, which may suggest good user satisfaction. However, it is important to note that user ratings may not always accurately reflect app quality. Therefore, the MARS was used in this study. The overall MARS score of the included apps was 3.2 of 5, which is considered acceptable. Considering the maximum and minimum MARS scores, substantial variability was observed across domains, including aesthetics, which had a range from 1.0 to 5.0. This variance may indicate a significant diversity in app quality, with some evidence suggesting the presence of low-quality apps currently available in the market. However, lower MARS scores could potentially be attributed to reviews of freely available app features.

The review and analysis of mobile app quality for common chronic diseases is crucial for future mHealth app development, as poor app quality can limit their effectiveness in health management [37]. Research on the quality of mHealth apps in Southeast Asia is limited, and the existing studies have primarily focused on COVID-19–related apps rather than those related to chronic disease management [38,39]. Therefore, the authors compared the MARS results with health care app studies from other countries. Our findings align with an assessment of nutrition-related mHealth apps in Korea, where the majority received an average rating, with a mean score of 3.28 of 5 [40]. In contrast, health apps designed for behavioral change in Denmark achieved a slightly higher average quality score, with an average MARS score of 3.48 of 5 [26]. Consistent with previous studies, our study revealed that functionality was best rated (3.9/5), whereas engagement consistently received the lowest score, with the mean score being 2.9 of 5 [41,42]. This indicates that the app developers focused on the functionality of the apps as an essential element in delivering outstanding experiences to the users. On the other hand, some studies discovered that information scored the lowest or fell into the low-to-moderate category [43-45]. This discrepancy highlights that the mHealth apps in the market need special attention to provide more advanced and effective features and capabilities for health management. Importantly, our results indicate that the key areas for improvement in mHealth apps are engagement and information. App developers should prioritize these domains, focusing on customization, interactive information delivery, and the integration of prompts such as feedback and reminders. Additionally, enhancing sharing functionality and offering more evidence-based content, engaging visuals, and data-driven information for users would improve the overall mHealth app quality.

From a health management perspective, mHealth interventions offer a significant opportunity to facilitate the monitoring of chronic conditions and improve self-management skills [14,24,26]. Evaluating the quality of mHealth apps can help us identify their positive impact on health and behavior outcomes among people with chronic diseases. According to Ryan and Sawin [46], who described an individual and family self-management theory, successful self-management should involve three key components: (1) individual competence, (2) individual motivation, and (3) social factors. mHealth apps have the potential to bridge these factors together to facilitate health management. Additionally, mobile technology promises to enable real-time remote monitoring systems and prompt feedback systems to improve health management [12]. App developers and users should be alert, as our findings show that most apps available in the market are lacking such components.

### Strengths and Limitations

The strengths of this study included that the findings were derived and evaluated from the clinical point of view, the study used a validated tool to determine the quality of mHealth apps, and the results can publicly provide review data to users as well as further direction for mHealth app development. This study has several identified limitations. First, we conducted a comprehensive review solely of the apps’ free features, excluding premium features due to budget constraints. Second, at the time the study was completed, it is possible that new mHealth apps or updated features had been released, which we were unable to consider. Third, the review was limited to specific common NCDs, and we could not provide an overview of other diseases. Fourth, our focus was on English-language apps available for download in commonly used mobile app stores, which might limit the generalizability of our findings. According to the world’s largest ranking of countries and regions by English skills, Malaysia ranks among the top 3 in the English Proficiency Index among Asian countries [47]. Nevertheless, we acknowledge that the focus on English-language apps could result in some percentage of Malaysians being left out. Importantly, app developers and researchers are increasingly recognizing the need to cater to diverse linguistic needs within mHealth apps to address the cultural and language diversity of countries like Malaysia [48,49].

### Future Direction for mHealth App Development

The findings of this study reveal that the current mHealth app market for managing NCDs in Malaysia is still in its nascent stages and is marked by a shortage of high-quality mHealth apps. In contrast to a previous study conducted in Malaysia in 2017, which predominantly featured informational apps [50], the market is currently undergoing a shift. It is now pivoting toward health management apps. This changing trend underscores the potential for mHealth technology to serve as a cornerstone in the management of chronic diseases in the future. For instance, the results of a prior study that specifically examined hypertension indicated that health care apps could serve as valuable additions to conventional treatment methods [51]. To effectively address NCDs, mHealth apps should incorporate self-monitoring capabilities, such as health tracking, goal setting, and personalized feedback. Additionally, app developers should emphasize elevating the overall quality of their apps by incorporating a variety of perspectives, including input from relevant health professionals and the integration of scientific evidence. It is essential to include this information in the app description to establish trust among users.

### Conclusion

A search for mHealth apps for common NCDs available in the Google Play Store and the Apple App Store revealed that most apps focused on weight management, followed by hypertension and type 2 diabetes mellitus. Self-monitoring features such as weight trackers, diet trackers, and step trackers are the core functions of current mHealth apps. This review also highlights the current market’s lack of evidence-based mHealth apps designed specifically for the self-management of chronic diseases. The lack of multidisciplinary teams in app development and health management was observed in the app stores. Evidently, the quality of mHealth apps currently available in the market should undergo ongoing assessment and enhancement to optimize their benefits for users in the realm of health management. Reviews of these apps can offer valuable insights to researchers, health care providers, and app developers, aiding them in delivering high-quality apps for effective health management. App developers and public health authorities should prioritize the development of evidence-based mHealth apps to enhance the mHealth ecosystem for users. Future studies on mHealth content analysis and app evaluation should encompass a broader spectrum of diseases, aiming for a more comprehensive approach that benefits diverse populations.

## Supplementary material

10.2196/49055 Multimedia Appendix 1 Assessment of the functionality of selected mobile health apps.

## Abbreviations

MARS: Mobile App Rating Scale
mHealth: mobile health
NCD: noncommunicable disease
NHMS: National Health and Morbidity Survey
WHO: World Health Organization
