# Supplementary material for: Mobile Apps for Common Noncommunicable Disease Management: Systematic Search in App Stores and Evaluation Using the Mobile App Rating Scale
Source: JMIR Mhealth Uhealth. 2024 Mar 12;12:e49055. doi: 10.2196/49055 (PMC11004629; doi:10.2196/49055)
Supplement: Multimedia Appendix 1 [file mhealth-v12-e49055-s001.docx]

**Appendix 1.** **Assessment of the Functionality of Selected mHealth Apps**

|  |  | Multidisciplinary team involvement (app development) | Health professional involvement | Educational content (e.g. blog) | Online consultation with health professional | Nutrient: Goal setting | Exercise: Goal setting | Steps: Goal setting | Goal setting: Weight | Weight tracking | Diet Tracking | Water intake tracking | Exercise tracker | Steps tracking | Bluetooth device connection | Social support | Health Calculator | Monitor Blood pressure | Monitor glucose | Monitor heart rate |
| --- | --- | --- | --- | --- | --- | --- | --- | --- | --- | --- | --- | --- | --- | --- | --- | --- | --- | --- | --- | --- |
| 1 | MyFitness Pal: Calorie Counter | x | / | / | x | / | / | / | / | / | / | x | / | / | x | / | x | x | x | x |
| 2 | Health & fitness tracker with calorie counter | x | x | x | x | / | x | x | / | / | / | / | x | / | x | x | / | x | x | x |
| 3 | Lifesum: Healthy eating & diet | x | / | x | x | / | x | x | x | / | / | / | x | x | x | x | / | x | x | x |
| 4 | Withings Health Mate | x | x | x | x | x | x | x | x | / | x | x | x | x | / | x | x | / | x | x |
| 5 | Fitbit | x | x | x | x | x | / | / | / | / | / | / | / | / | / | x | x | x | x | x |
| 6 | HealthifyMe | x | / | / | / | / | x | / | / | / | / | / | x | / | / | x | x | x | x | x |
| 7 | Noom: Weight loss | x | / | / | / | / | x | / | / | / | / | / | x | / | x | x | x | / | / | x |
| 8 | Personal Health Monitor | x | x | x | x | x | x | x | x | / | x | x | x | x | x | x | x | / | x | x |
| 9 | Life Extend: Healthy Habits | x | x | / | x | x | / | x | x | / | / | x | / | x | / | / | x | x | x | x |
| 10 | Qardio Heart Health | x | x | x | x | x | x | x | x | / | x | x | x | x | / | / | x | / | x | / |
| 11 | FitTrack MyHeath: Track Scale | x | x | x | x | x | x | x | / | / | / | x | x | x | / | x | x | x | x | x |
| 12 | Calorie counter by lose it ! | x | x | x | x | / | / | x | x | / | / | x | / | x | / | / | x | x | x | x |
| 13 | One Drop: Better Health Today | x | / | / | / | x | x | x | x | / | / | x | x | x | x | x | x | x | / | x |
| 14 | Calorie Counter - MyNetDiary | x | / | / | / | / | x | / | x | / | / | / | x | / | x | x | x | x | x | x |
| 15 | Healthi: Personal Weight Loss | x | / | x | x | / | x | x | x | / | / | x | x | x | x | / | x | x | x | x |
| 16 | Health Diet Foods Fitness Help | x | / | / | / | x | x | x | x | x | x | x | x | x | x | x | x | x | x | x |
| 17 | Unimeal: Healthy Diet&Workouts | x | / | / | x | / | x | x | x | / | / | / | / | x | x | x | x | x | x | x |
| 18 | Health Click Away | x | / | x | / | / | x | / | / | / | / | / | / | / | / | / | / | x | x | x |
| 19 | Possible-Nutrition Weight Loss | x | / | / | / | x | x | x | / | / | / | / | x | x | x | / | x | x | x | x |
| 20 | Health Club-Home workouts& Fitness-calorie tracker | x | x | / | x | x | x | x | x | / | / | / | / | x | x | x | x | x | x | x |
| 21 | Smart Diet Planner weight loss | x | / | x | x | / | x | x | / | / | / | x | x | x | x | x | x | x | x | x |
| 22 | Heart Care Health & Diet Tips | x | / | / | / | x | x | x | x | x | x | x | x | x | x | x | x | x | x | x |
| 23 | Calorie Counter + (Nutracheck) | x | x | x | x | x | x | x | / | / | / | x | x | x | x | x | x | x | x | x |
| 24 | Health Mate - Calorie Counter & Weight Loss App | x | x | x | x | / | x | x | / | / | / | / | x | x | x | x | / | x | x | x |
| 25 | Doctor2u- One Stop Healthcare | x | / | x | / | x | x | x | x | x | x | x | x | x | x | x | x | x | x | x |
| 26 | BookDoc- Go Active Get reward | x | / | x | / | x | x | x | x | / | x | x | x | x | / | x | x | / | / | / |
| 27 | Health Pal - Fitness, Weight loss coach, Pedometer | x | x | x | x | x | x | / | / | / | / | / | / | / | x | x | / | x | x | x |
| 28 | Creda- manage chronic condition | x | / | x | x | / | x | x | / | x | / | x | x | x | x | x | x | / | / | / |
| 29 | Mhealth | x | x | x | x | x | x | x | / | / | x | x | x | x | / | x | x | x | x | / |
| 30 | my Mhealth | x | / | / | x | x | x | x | x | x | x | x | / | x | x | x | x | / | / | x |
| 31 | Zero: Fasting & Health Tracker | x | / | / | x | x | x | x | / | / | / | x | x | x | x | x | x | x | / | / |
| 32 | Lose Weight at Home in 30 Days | x | x | x | x | x | x | x | / | / | x | x | / | x | x | x | x | x | x | x |
| 33 | BodyFast Intermittent Fasting | x | / | / | / | x | x | x | / | / | x | / | x | x | x | x | x | x | x | x |
| 34 | BetterMe: Health Coaching | x | / | / | / | x | x | x | x | / | / | x | / | x | x | x | x | x | x | x |
| 35 | Weight Loss Running by Slimkit | x | x | / | x | x | x | / | / | / | x | x | x | / | x | x | x | x | x | x |
| 36 | My Diet Coach - Weight Loss | x | x | x | x | / | x | x | / | / | / | x | x | x | x | x | x | x | x | x |
| 37 | Fitness Coach & Diet: FitCoach | x | / | x | x | x | / | x | / | / | / | x | x | x | x | x | x | x | x | x |
| 38 | Argus: Calorie Counter & Step | x | x | x | x | / | x | x | x | x | / | x | / | x | / | / | x | x | x | / |
| 39 | Speedoc - Care Comes to You | x | / | / | / | x | x | x | x | x | x | x | x | x | x | x | x | x | x | x |
| 40 | Glucose Buddy Diabetes Tracker | x | / | x | / | x | x | / | x | / | / | x | x | / | x | x | x | / | / | x |
| 41 | DOC2US - Trusted Online Doctor | x | / | / | / | x | x | x | x | / | x | x | x | x | x | x | x | / | / | / |
| 42 | Foodvisor - Nutrition & Diet | x | / | / | / | / | x | x | / | / | / | / | / | x | x | x | x | x | x | x |
